# Hax-1 Regulates Radiation-Induced Mitochondrial-Dependent Apoptosis of Uveal Melanoma Cells through PI3K/AKT/eNOS Pathway

**DOI:** 10.1155/2022/2956888

**Published:** 2022-05-13

**Authors:** Sha Wang, Jia Tan, Lu Chen, Jinwei Wang

**Affiliations:** ^1^Eye Center of Xiangya Hospital, Central South University, 87 Xiangya Road, Changsha, China 410008; ^2^Hunan Key Laboratory of Ophthalmology, 87 Xiangya Road, Changsha, China 410008

## Abstract

Uveal melanoma is an aggressive skin cancer that remains insurmountable and is accompanied by inferior prognostic results. The proliferative and survival mechanisms of uveal melanoma cells need to be further investigated to improve the treatment of uveal melanoma. According to reports, HAX-1 is an antiapoptotic protein vital for multiple malignancies. Nevertheless, the role and causal link of HAX-1 in uveal melanoma are still elusive. The survival diversity of uveal melanoma sufferers with diverse haX-1 expressing levels was studied by TCGA database. Patients in the risk_high_ group exhibited greater levels of HAX-1 in contrast to the risk_low_ group, and individuals with higher HAX-1 levels displayed inferior survival times. The outcomes of CCK-8 and clonogenesis revealed that the proliferative rate of haX-1 knockout cells was slower. The result of scratch experiment shows that the ability of scratch recovery after HAX-1 is reduced. Transwell migration and tumor cell pelletization experiments showed that siHAX-1 significantly reduced cell migration and tumor cell pelletization. After haX-1 was knocked out, the loss of MMP was decreased, the transfer of CyT C was elevated, and the protein expression of Bax, Caspase 3, and Bcl2 was elevated, suggesting that mitochondria-induced apoptosis was increased. Sihax-1 treatment remarkably decreased the phosphonation of phosphatidylinositol 3-kinase (PI3K)/AKT/mammalian target of rapamycin (mTOR)/endothelial NO synthase (eNOS) in mum-2B and C918. Pretreatment with LY294002 significantly restored iHAX-1-induced decline in PI3K/AKT/mTOR/eNOS phosphorylation. Therefore, our results suggest that haX-1 induces radiation-dependent apoptosis of UM cells via the PI3K/AKT/eNOS signal path.

## 1. Introduction

UM is one of the most seen primary intraocular malignancies in adults. It is mainly derived from uveal melanocytes and has the features of high proliferation activity and easy metastases [1, 2]. The prevalence of melanoma rises incessantly in many nations and has become one of the major causes of tumor-associated incidence and death across the globe [3]. Due to the special structure of the eye, the initial tumor symptoms are not obvious, and the patient's attention is not paid attention to. This has caused many patients with liver or systemic metastases at the time of diagnosis, which often leads to higher mortality [4, 5]. The treatment methods of UM mainly include eyeball enucleation, local tumor resection, local radiotherapy (external scleral application radiotherapy, stereo radiotherapy, and proton beam therapy), and laser photocoagulation therapy (transpupillary thermotherapy and photodynamic therapy) [6, 7]. At present, extrascleral application radiotherapy is a more effective method for the treatment of UM, which can not only increase the effective transmission speed of radiation but also reduce the damage of radiation to normal tissues [8, 9]. As current treatment methods still face challenges in improving patients' clinical survival and visual function, studying the molecular mechanism of UM is imperative for early diagnosis and ameliorating the long-term prognosis of patients.

Apoptosis is one of the methods of programmed cell death (CD), and it is vital for the elimination of impaired cells [10]. Apoptotic events have evident morphology and biochemistry variations and are pivotal for the growth and developmental process of organs and tissues, immunity, metabolism, and the elimination of abnormal cells [11]. Caspase is a protease that promotes cell apoptosis and plays a central role in the network of cellular apoptotic mechanisms [12]. Researches have shown that Caspases can induce cell apoptosis in three main ways: (1) death receptor pathway (exogenous) apoptosis, (2) mitochondrial pathway (endogenous pathway), and (3) internal apoptosis of the plasma reticulum stress pathway [13, 14].

The key effects of mitochondria on apoptotic events have been broadly revealed [15]. In the process of apoptotic events, the permeability of the mitochondrial membrane increases, releasing soluble mitochondrial membrane interstitial proteins and further destroying the cell structure. Among these lethal proteins, some (Cyt c, Smac/DIABLO, Omi/HtrA2, etc.) can activate caspases, while others (endo G, AIF, Omi/HtrA2, etc.) act in a non-caspase-dependent manner. The releasing of those proteins is the result of the destruction of the completeness of the mitochondria OM via permeabilisation [16, 17]. The kinetic events in mitochondria eventually decide the onset of apoptotic events, highlighting the tight association between mitochondria function disorder and CD. In addition, Bcl-2 family protein is also vital for the occurrence of apoptotic events. Bcl-2 family members modulate the mitochondria apoptosis signal path via regulating the permeation of the mitochondrial OM. Upon apoptosis stimulation, Bax/Bak translocates to the mitochondrial membrane, promotes the releasing of Cyt c from the inner mitochondrial membrane space into the cytoplasm, and induces the occurrence of cell apoptosis [18]. HS-1 related protein-1 (HAX) -1) is a +35 kDa protein, found everywhere in mitochondria [19]. On the foundation of its low sequencing homology with Nip3 and structure similarity with Bcl-2 family protein, as a mitochondrial antiapoptotic protein, HAX-1 is considered to participate in apoptotic events or programmed CD regulation, and its abnormal expression is related to many serious diseases, including neurodevelopmental delay, cancer, and cardiovascular disease [20, 21]. A report pointed out that HAX-1 can regulate the cell death process in myocardial ischemia-reperfusion injury through ERS and mitochondrial stability [22]. Recently, a research showed that the decomposition of HAX-1 induced CD in mankind B-cell lymphomas, confirming the critical effects of HAX-1 on regulating cellular survival [23]. Another study pointed out that the abnormal expression of HAX-1 protein is vital for suppressing the apoptosis of glioblastoma cells [24]. Yan et al. revealed that HAX-1 can suppress the apoptotic events of prostate oncocytes via the inactivation of yellow membrane-9 [25]. According to Oncomine, the tumor microarray database, the expression of HAX-1 is high in many diseases like lung carcinoma, lymphoma, melanoma, and myeloma [26]. However, the molecular mechanism of HAX-1's effect on uveal melanoma has not been studied.

Here, our team was the first to reveal that HAX-1 knockout affects the viability, migration, and tumor cell spheroidizing ability of UM cells. The effects of HAX-1 on mitochondrial-dependent induction of uveal melanoma cell apoptosis are caused by activating the PI3K/AKT/eNOS signal path and favorable modulation of Bax, caspase 3, and Bcl2.

In this research, the TCGA database was employed to study the survival differences of patients with uveal melanoma with diverse expression levels of HAX-1. Our team found that the expression of HAX-1 in the risk_high_ group was greater in contrast to the risk_low_ group, and the survival duration of patients with higher HAX-1 levels was inferior. In addition, we also discovered that HAX-1 participates in the modulation of uveal melanoma cellular viability, metastasis, and tumor ring formation via modulating PI3K/AKT/eNOS and triggers UM cell apoptosis via mitochondria dependence. For that reason, the present research primarily discusses the expressing features of HAX-1 in uveal melanoma and the causal link affecting cell apoptosis.

## 2. Methods

### 2.1. Data Collection

Download RNA-seq data of uveal melanoma patients from TCGA database and relevant clinical data of patients. The extracted clinical data included overall survival time (OS.time), age, sex, and IDH gene mutation status. Data from 88 patients with uveal melanoma were extracted by matching the samples with RNA-SEQ data, CNV data, and relevant clinic information for analysis.

### 2.2. Differential Analysis of the Expression Profile of UM Patients with Different HAX-1 Expression

For 88 UM expression spectrum data in TCGA, HAX1_H:40 and HAX1_L:40 were used as grouping basis. DESeq2 package was used for difference analysis and screen *P* < 0.05 and absolute value Log2(fold change) > 1 as the significant gene for difference. Finally, the difference genes were shown by volcano map. Differentially expressed genes in heat map were stratified and clustered. The correlation between HAX1 gene expressing and OS rate was analyzed by univariable Cox based on the clinical information data of UM in TCGA database.

### 2.3. Differential Gene GO Analysis and KEGG Pathway Analysis

TCGA expression profile chip was corrected, edgeR of R language was used for differential gene analysis, and pheatmap package was used to draw differential gene volcano map and cluster analysis heatmap. The screening conditions were logFC ≥ 1 or ≤-1, and *P* < 0.05 had significance on statistics. DAVID online program and clusterProfiler package were employed to study the differentially expressed EC genes. Finally, Gene Ontology (GO) analysis with FDR < 0.05 was selected as the result of enrichment function, and the GGploT2 package of R language was used for mapping. KEGG pathway analysis is functionally classified and enriched by a hypergeometric distribution. The dataset was analyzed by KEGG pathway via the “Limma” R package for differential analysis.

### 2.4. Subsistence Analysis

The Kaplan-Meier survival curve was drawn, and logrank rank-sum test was employed to evaluate the overall survival of patients in the risk_high_ group and risk_low_ group. ROC curves were employed to evaluate the prediction power of the prognostic risk model at 1, 3, and 5 years of survival, and heat maps of the risk_high_ group and risk_low_ group were drawn. The univariable and multivariable Cox regressive analyses were employed to evaluate the correlation of clinical variables and risk scores with patient prognoses. The pictures were drawn using R software and SPSS 22.0 (IBM, Armonk, NY, USA).

### 2.5. MUM-2B and C918 Cells and their Cultivation

UM lineage cells Mum-2B and C918 were bought from SICB, CAS. The uveal melanoma lineage cells mum-2B and C918 were cultivated in DMEM intermediary with 10% serum, and 1% PNC/Streptomycin double antibody solution was added into the medium. The cell incubator temperature was set at 37°C and CO_2_ content was 5%. The fresh medium was replaced every 2 days. When the medium was replaced, the cell surface was washed with PBS solution to remove some metabolic substances secreted by cells. When the cells adhered to the wall and grew to 80%~90%, 0.25% trypsin was added for digestion and passage of cells. Stable and well-growing third-generation melanoma cells were collected for subsequent experimental operations.

### 2.6. Synthesis of HAX-1 siRNA

According to the design principle of siRNA sequence and according to the sequence of HAX-1 gene (no. NM006118) in GenBank database, the 540-640 nucleotide of CDS sequence was selected as siRNA sequence, and this sequence was compared with the homology of other genes and EST sequences in NCBI database, which confirmed that there was no homology with other genes and EST sequences. The following two haX-1 siRNA target sequences with BglII and HindIII sticky ends were designed and synthesized:

5,—CATCCCCAACCAGAGAGGACAATGATCTTTCAAGAGAAGATCATTGTCCTCTCTGGTTTTTTTA—37

5,—AGCTTAAAAAAACCAGAGAGGACAATGATCTTCTCTTGAAAGATCATTGTCCTCTCTGGTTGGG—37

### 2.7. Cell Proliferation Detected by CCK8 Method

The cells from each group were digested by trypsin to prepare cell suspension and inoculated on 96-well dishes with inoculation density of 5 × 103/well. The cells were continued to be cultured at 37°C, and 100 *μ* L CCK 8 liquor was supplemented into all wells at 0 h, 24 h, 48 h, and 72 h, separately, and cultivated under 37°C for 30 min under dark conditions. The OD result of all wells at 450 nm was measured on a multifunctional micro plate analyzer, and the cellular activity (%) − (experiment group optical density/control group optical density) × 100% was calculated. Three multiple holes were set at each time point in every group, and the assay was performed in triplicate.

### 2.8. Clone Formation Experiment

Mum-2b and C918 cells were seeded to 6-well dishes with about 500 cells in each well and grouped according to Method 1.3. After 7 days of culture, the supernatant was discarded, 4% paraformaldehyde was subjected to fixation for 20 min, and 0.1% gentian violet was dyed for 15 min. After washing and drying, the number of clones formed was observed under a microscope, and the clone forming rate was computed. Cell clone forming rate (%) = overall cell clones/seeded cells × 100%.

### 2.9. Scratch Healing Test

Mum-2b and C918 cells were seeded into 6-well dishes, and 1 × 106 cells were inoculated in every well and cultured to 90%-100% fusion degree. The bottom of 6-well plates was gently scratched with 200 *μ*L spear head, a vertical line and a horizontal line were drawn, and the cells were cleaned with PBS for two times. The 6-well plate was observed under an inverted microscopic device. Images were captured near the junction of vertical and horizontal lines and taken again at the same position 24 hours later. Use ImageJ software to measure the scratch width, mobility/% = (0 h scratch width − 24 h scratch width)/0 h scratch width × 100%. Each dosing group was set with 3 multiple wells, and the assay was independently performed in triplicate.

### 2.10. Transwell Assay Detected by Cell Migration

Matrigel matrix adhesive was diluted 9 : 1 in precooled culture medium, and 40 *μ*L Matrigel diluent was added to each well in the upper chamber of Transwell chamber and cultivated under RT for 5 h. Mum-2b and C918 cells of logarithmic growth uveal melanoma cells were precooled and washed with PBS, subjected to digestion by 0.25% trypsin, and then added with culture medium without fetal bovine serum to prepare single cell suspension (5 × 104 cells/mL). 200 *μ*L single cell suspension was supplemented to each well of the upper chamber. 600 *μ*L culture intermediary with 10% FBS was supplemented into each well in the lower chamber, cultivated for 24 h under 37°C within an incubating device at 5% carbon dioxide, cleaned in PBS, subjected to fixation in PFA for 10 min, dyed in 0.1% gentian violet for 10 min, and wiped with cotton swabs for nonmigrated cells. The number of transplanted cells was observed under microscope.

### 2.11. Flow Cytometry Apoptosis Detection

The cells were inoculated on 6-well dishes and apoptotic events were identified via flow cell technique using Annexin V-FITC/PI dyeing (Nanjing KGI Biotechnology Development Co., Ltd.). Flow cytometry apoptosis detection is as follows: 24 h posterior to transfection, the cells were digested by trypsin and harvested and then suspended and washed with 100 *μ*L 1×Binding Buffer (Shanghai Biyantian Biotechnology Co., Ltd.) for each tube. Annexin V-APC 5 *μ*L reagent was added and incubated for 15 min. Apoptotic events were identified via flow cell technique at 4°C.

### 2.12. Western Blot Detection

The cells were harvested 3648 h posterior to transfection and subjected to centrifugation at 600 r/min for 3 rains, and the supernate was removed. The cells were cleaned with 5 mL ice precooled PBS for 2 times to collect cell precipitates. 200 *μ*L single detergent lysis solution containing protease inhibitor was supplemented into a 60 mm diameter cultivation plate, followed by an ice bath of about 18 rains and centrifugation of 13000 r/min for 10 rains. The supernate was taken, and the total protein was quantitatively determined and moved onto PVDF film by SDS-PAGE. The cells were sealed with 5% skim milk powder, sealed with primary antibody (1 : 1000, Protein Tech Group, USA) under 4°C nightlong, and cleaned three times in TBST, 300 s each. The second antisubstance (1 : 500, Protein Tech Group, USA) was incubated under RT for 60 min and afterwards cleaned three times in TBST, and ECL chemiluminescence was performed. FluorChemFC2 imager from CELLBIOSCIENCES was used for luminescence development.

### 2.13. Statistical Analysis

Using R (V3.6.1) and SPSS 20.00, the univariable Cox regressive analyses were completed on the expressing level of HAX-1 and overall survival in UM clinical case data. Statistical tests were conducted by bilateral tests. *P* < 0.05 had significance on statistics.

## 3. Results

### 3.1. Differential Analysis of the Expression Profile of HAX-1 Overexpression and Normal Expression in Uveal Melanoma

**Figure 1:**
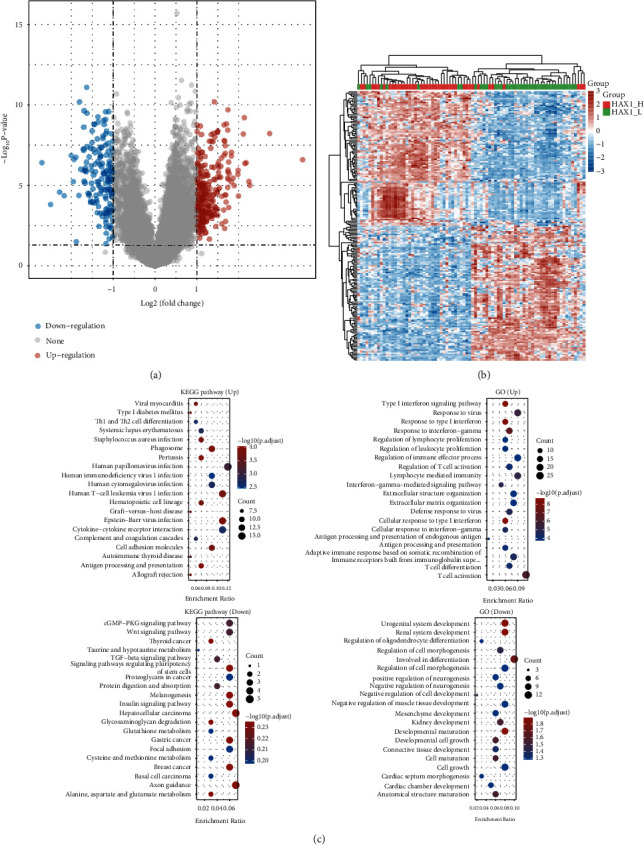
Difference analyses of HAX-1 overexpression and normal expression in patients with uveal melanoma. (a) TCGA volcano map of differentially expressed genes in patients with uveal melanoma in different survival periods. (b) Layer clustering thermograph of differential genes. (c) Enrichment analysis of the upregulated and downregulated genes GO and KEGG in the survival group of patients with uveal melanoma in TCGA.

Raw counts and corresponding clinical information of RNA sequence (level 3) from 80 UVM tumors were acquired from TCGA dataset. Using the high and low expression of HAX1 as grouping basis (HAX1_H:40 and HAX1_L:40), Limma software of R program was employed to explore the differentially expressed mRNA. According to the screening criteria modified *P* < 0.05 and absolute value Log2(fold change) > 1. A total of 407 mRNA genes were screened, including 252 upregulated genes and 155 downregulated genes (Figure 1(a)), and top 20 upregulated genes and top 20 downregulated genes are shown in Table 1.  Figure 1(b) thermograph shows layer clustering of expressing levels of DEGs. To identify the potential capabilities of underlying targets, the data was studied through feature enrichment. GO is a extensively utilized method to annotate functional genes, particularly MF, BP, and CC. KEGG enrichment analysis is useful for analyzing gene function and related high-level genomic function data. To further reveal the carcinogenic effects of targeted genes, the clusterProfiler package in R was employed to study the GO function of underlying mRNAS and realize the KEGG pathway enrichment. Cytokine enriching assay revealed that the upregulated genes were mainly distributed in viral arditis, type 1 diabetes mellitus, Th1 and Th2 cellular differentiation, systemic lupus erythematosus, SA infection, phagosome, pertussis, human papillomavirus infection, HIV-1 infection, and HCMV infection. KEGG pathway analyses revealed that downregulated genes were mainly distributed in Wnt signal path, thyroid cancer, TGF-beta, signal path, signal paths modulating pluripotency of stem cells, proteoglycans in carcinoma, etc. GO term enrichment outcomes revealed that the upregulated genes were primarily distributed in type I interferon signaling pathway, reaction to viruses, reaction to type I IFN, reaction to IFN-*γ*, modulation of lymphocyte proliferation, and other pathways. GO term enrichment showed developmental maturation, developmental cell growth, connective tissue development, cell maturation, cell growth, cardiac septum morphogenesis, cardiac chamber development, and packet structure Hood isotherm pathway (Figure 1(c)).

### 3.2. Cox Analysis of the Correlation between HAX-1 Expression and Overall Survival

**Figure 2:**
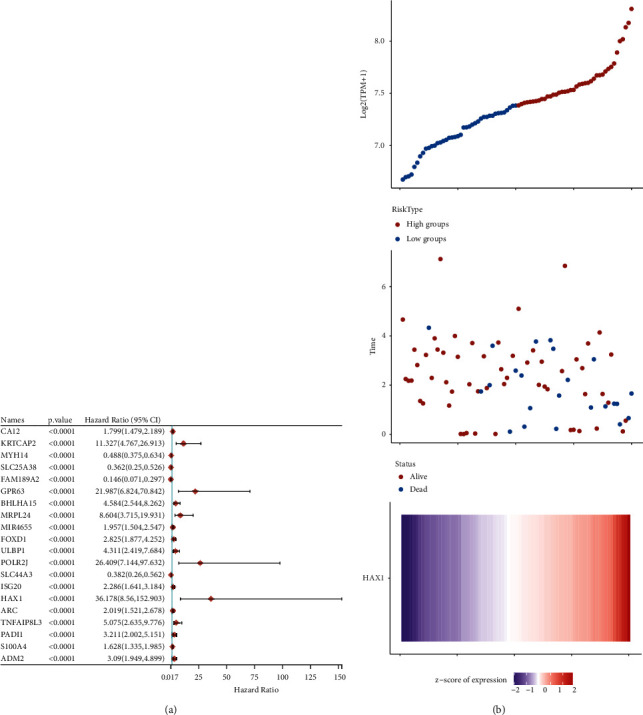
Cox analysis of correlation between haX-1 expressing and OS. (a) Forest map of 20 differentially expressed genes related to OS in whole-gene Cox regressive analysis. (b) Risk factor association diagram of differential expression gene prognostic model. Above: risk_high_ (red) and risk_low_ (blue) in a prognostic model. Risk score distribution of uveal melanoma patients. Middle image: scatter plot shows the survival of patients with GBM in the model. Red dots are patients who died and blue dots are patients who survived. Figure below: a calorimetric map of the genetic expression of haX-1 in the model.

To further study the effect of HAX-1 on the prognosis of uveal melanoma, the DEGs were obtained, univariate Cox regression analysis was performed, and forest maps were drawn. As shown in Figure 2(a), the Cox risk regression analysis identified 20 optimal differentially expressed genes. As you can see from the risk factor association graph, there were significantly more deaths and fewer survivors in the risk_high_ group. In addition, the expressing level of haX-1 was greater in the risk_high_ group in contrast to the risk_low_ group (Figure 2(b)). K-M survival analyses were used to evaluate the OS of sufferers in diverse groups. The OS of sufferers in the risk_low_ group (blue) was remarkably higher in contrast to the risk_high_ group (red) (*P* < 0.05) and the difference was statistically significant (Figure 2(c)). On the foundation of the prognosis gene model, the overall survival rate of 1, 3, and 5 years in the future was predicted by ROC curve. The results showed that the constructed model exhibited satisfactory prediction capability (Figure 2(d)).

### 3.3. HAX-1 Knockout Affects UM Cell Viability, Migration, and Oncocyte Spheroidizing Ability

**Figure 3:**
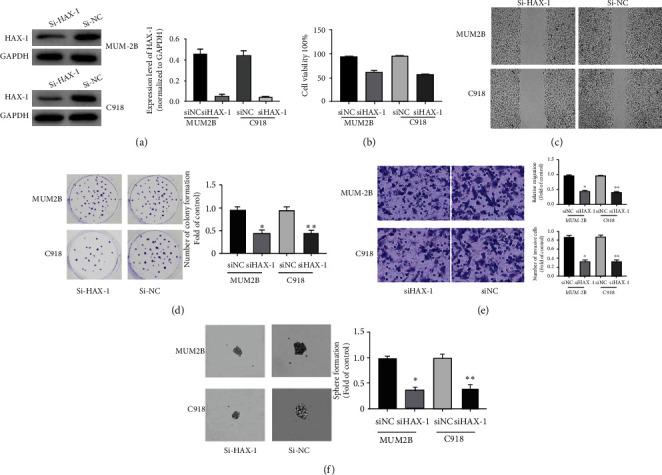
HAX-1 knockout affects the viability and migration of UM cells and the ability of tumor cells to form balls. (a) WB assay of HAX-1 protein expressing levels in uveal melanoma lineage cells MUM-2B and C91. (b) CCK-8 method was employed to identify the proliferative effect of transfected siHAX-1 and si control on MUM-2B and C91 cells. siHAX-1 vs. control group, ^∗^*P* < 0.05 and ^∗∗^*P* < 0.01. (c) Scratch test to assess the effect of HAX-1 on the migration of MUM-2B and C91 cells. (d) The clone formation experiment detects the effect of HAX-1 on the proliferative ability of MUM-2B and C91 cells. siHAX-1 vs. the controls, ^∗^*P* < 0.05 and ^∗∗^*P* < 0.01. (e) Migration test (using Matrigel Transwell chambers) is used to study cell migration. siHAX-1 vs. the controls, ^∗^*P* < 0.05 and ^∗∗^*P* < 0.01. (f) Tumor sphere formation ability experiment to assess the roles of HAX-1 in the tumor sphere formation capability of MUM-2B and C91 cells.

To better explore the roles of HAX-1 in UM cells, our team used chemically synthesized siRNA to knock down haX-1 expression in mum-2B and C918 cells. At 48 h after transfection, WB was employed to evaluate the efficiency of siRNA knockout. The outcomes showed that HAX-1 siRNA effectively reduced the protein expressing level of HAX-1 in mum-2B and C918 cells (Figure 3(a)). By CCK-8 detection, we found that cell proliferation rates of Mum-2B and C918 cells subjected to siRNA treatment were remarkably lower in contrast to those subjected to siRNA treatment (Figure 3(b)). In scratch experiments, siHAX-1's ability to recover scratch was remarkably improved in mum-2B and C918 cells compared with siRNA control cells (Figure 3(c)). Results of clone forming assays revealed that siHAX-1 remarkably reduced the quantity of colony formation in soft AGAR (Figure 3(d)). In Transwell migration experiment, siHAX-1 significantly reduced cell migration in mum-2B and C918 cells in contrast to siRNA control cells (Figure 3(e)). In addition, haX-1 knockout significantly reduced the pellet-forming ability of UM cells in contrast to the controls (Figure 3(f)). Those results reveal that HAX-1 knockout affects UM cellular activity, metastasis, and oncocyte pelletogenesis.

### 3.4. HAX-1 Induces Apoptosis in the Mitochondrial-Dependent Pathway

**Figure 4:**
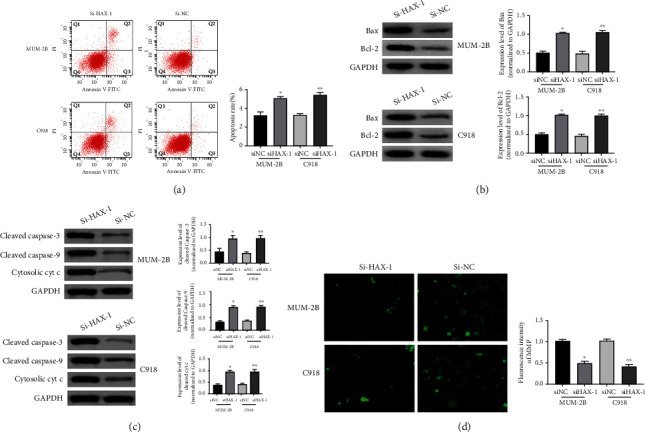
HAX-1 induces apoptosis in the mitochondrial-dependent pathway. (a) Flow cytometry to detect flow cytometry cycle distribution. (b) WB assay to identify Bax and Bcl-2 protein expressing levels in UM lineage cells MUM-2B and C91. (c) Western blot analysis to detect the expression levels of caspase-3/9 and cytosolic Cyt c protein in the uveal melanoma cell lines MUM-2B and C91. (d) Immunofluorescence detection of MMP expressing in UM lineage cells MUM-2B and C91.

We next examined whether HAX-1 triggered apoptosis in uveal melanoma cells. In apoptotic events identified via flow cell technique, siHAX-1 triggered programmed cell death in mum-2B and C918 cells in contrast to siRNA control cells (Figure 4(a)). In addition, siHAX-1 increased protein expressing levels of Bax mum-2B and C918 cells (Figure 4(b)). Stimulation of caspase-9, reduction of MMP, and transfer of Cyt c from the mitochondrion to cytosol can verify the occurrence of mitochondrial apoptosis. For that reason, to better verify the effects of mitochondria on haX-1-triggered apoptotic events, we examined variations in protein expressing levels of caspase-3/9 and cytochrome C levels. The outcomes revealed that siHAX-1 elevated the expression of Caspase-3/9 and Cytosol cyt C in mum-2B and C918 cells in contrast to the controls (Figure 4(c)). Moreover, siHAX-1 remarkably decreased MMP in mitochondrial pathways in mum-2B and C918 cells compared to the controls (Figure 4(d)). Those results reveal that HAX-1 triggers programmed cell death in uveal melanoma cells in a mitochondrion-reliant signal path.

### 3.5. HAX-1 Induces UM Cell Apoptosis through AKT/eNOS Signal Path

**Figure 5:**
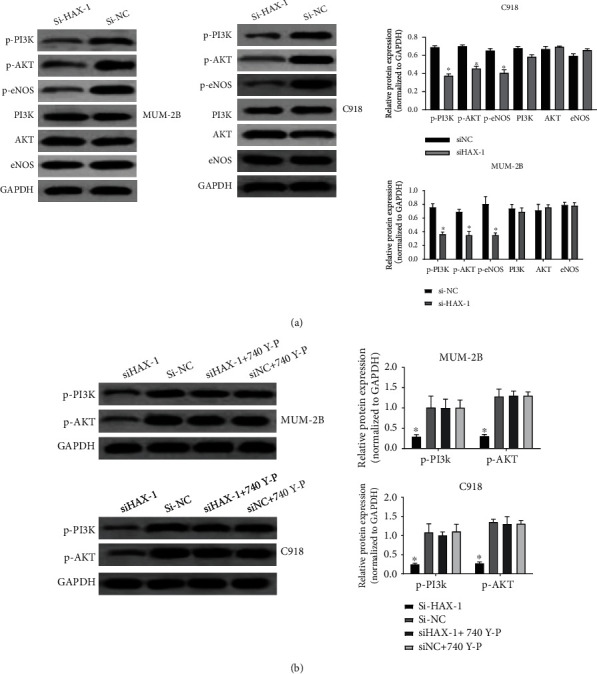
HAX-1 induces uveal melanoma programmed cell death via the AKT/eNOS signal path. (a) WB assay to identify the effect of HAX-1 on PI3K/AKT/mTOR/eNOS pathway. (b) WB assay to assess the roles of siHAX-1+740 Y-P in the expressing levels of phosphorylated PI3K and AKT proteins.

To investigate the causal link involved in the apoptosis-inducing role of HAX-1, WB was employed to identify the expression and phosphonation of PI3K/AKT/mTOR/eNOS. Treatment with SihaX-1 remarkably decreased the phosphonation of PI3K/AKT/mTOR/eNOS in mum-2B and C918 (Figure 5(a)). Pretreatment with 740-YP significantly restored the decrease in PI3K and AKT phosphorylation induced by SihaX-1 (Figure 5(b)). These data suggest that the apoptosis-inducing effect of SiHAX-1 in mum-2B and C918 cells might be under the mediation of the PI3K/AKT/mTOR/eNOS signal path.

### 3.6. HAX-1 Regulates UM Cell Viability, Migration, Oncocyte Spheroidization Ability, and Mitochondrial-Dependent Apoptosis by Regulating the AKT/eNOS Signal Path

**Figure 6:**
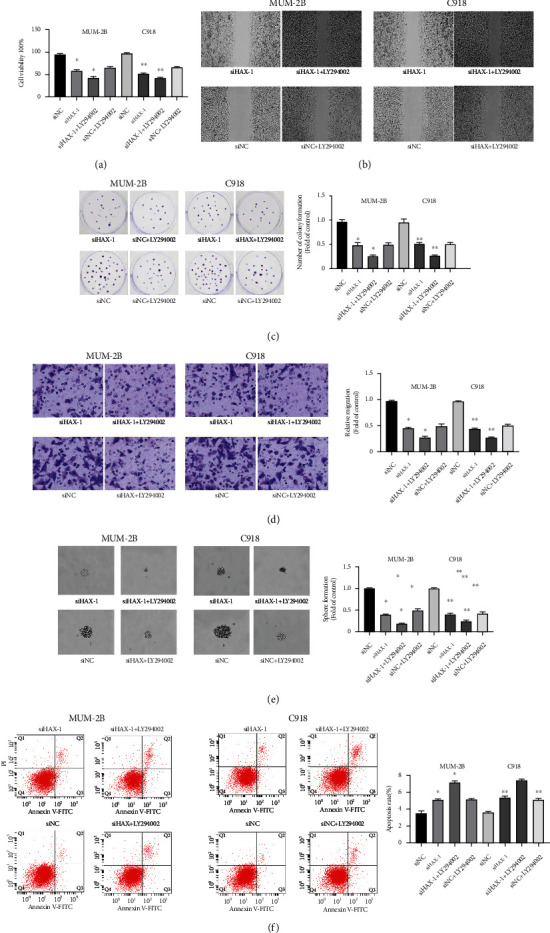
HAX-1 regulates uveal melanoma cell viability, migration, tumor cell spheroidization ability, and mitochondrial-dependent apoptosis by regulating the AKT/eNOS pathway. (a) CCK-8 method was employed to identify the proliferative effect of MUM-2B and C91 cells in siHAX-1+ LY294002 and siNC+LY294002 groups. siHAX-1 vs. the controls, ^∗^*P* < 0.05 and ^∗∗^*P* < 0.01; siHAX-1+LY294002 vs. siNC+LY294002, ^∗^*P* < 0.05 and ^∗∗^*P* < 0.01. (b) Scratch test to evaluate the effect of HAX-1+LY294002 on the migration of MUM-2B and C91 cells. (c) The clone formation experiment detects the effect of siHAX-1+LY294002 on the proliferation of MUM-2B and C91 cells. siHAX-1 vs. the controls, ^∗^*P* < 0.05 and ^∗∗^*P* < 0.01; siHAX-1+LY294002 vs. siNC+LY294002, ^∗^*P* < 0.05 and ^∗∗^*P* < 0.01. (d) Migration test (using Matrigel Transwell chambers) is used to study cell migration. siHAX-1 vs. control group, ^∗^*P* < 0.05 and ^∗∗^*P* < 0.01; siHAX-1+LY294002 vs. siNC+LY294002, ^∗^*P* < 0.05 and ^∗∗^*P* < 0.01. (e) Tumor spherule forming ability experiment to assess the role of siHAX-1+LY294002 in the tumor spherule forming capability of MUM-2B and C91 cells. siHAX-1 vs. the controls, ^∗^*P* < 0.05 and ^∗∗^*P* < 0.01; siHAX-1+LY294002 vs. siNC+LY294002, ^∗^*P* < 0.05 and ^∗∗^*P* < 0.01. (f) Flow cytometry experiment to assess the roles of siHAX-1+LY294002 in the apoptotic capability of MUM-2B and C91 cells. siHAX-1 vs. the controls, ^∗^*P* < 0.05 and ^∗∗^*P* < 0.01; siHAX-1+LY294002 vs. siNC+LY294002, ^∗^*P* < 0.05 and ^∗∗^*P* < 0.01.

It is known to all that the AKT/eNOS signal path is pivotal for the genesis and development of tumors. It is vital for cellular proliferation, differentiation, and cell viability modulation [27]. The gain or loss of function caused by abnormal expression of related genes and molecules in this pathway can lead to abnormal proliferation, apoptosis, and invasion of tumor cells [28]. Tumor progression is related to aberrant genetic stimulation in those signal paths as well, which might induce elevated cellular growth and survival [29]. Next, we examined whether haX-1 affects uveal melanoma cells through the AKT/eNOS pathway. 48 h after transfection, Western blot results showed that the decrease in PI3K and AKT phosphonation caused by HAX-1 knockdown was significantly restored by LY294002 in mum-2B and C918 cells (Figures 5(a) and 5(b)). By CCK-8 assay, we found that LY294002 preconditioning restored the decrease in mum-2B and C918 cellular proliferative rates caused by siHAX-1 treatment (Figure 6(a)). In the scratch experiment, LY294002 pretreatment restored the reduced scratch recovery ability of SIHAX-1 in mum-2B and C918 cells (Figure 6(b)). The results of clone formation experiments showed that LY294002 pretreatment restored the reduction in the number of colonies formed in soft AGAR caused by SiHAX-1 (Figure 6(c)). In Transwell migration experiment, pretreatment with LY294002 restored the decrease in cell migration induced by SIHAX-1 in mum-2B and C918 cells (Figure 6(d)). These results suggest that haX-1 knockout reduced uveal melanoma cell viability and migration ability reversed by LY294002. In addition, LY294002 also reversed the tumor-forming ability of uveal melanoma cells reduced by HAX-1 knockdown (Figure 6(e)), as well as the apoptosis of uveal melanoma cells induced by HAX-1 in the mitochondria-dependent pathway (Figure 6(f)). Those results reveal that haX-1 affects UM cell viability, the ability of migrating tumor cells to form pellets, and mitochondria-dependent apoptosis via the AKT/eNOS pathway.

## 4. Discussion

Uveal melanoma (UM) is a commonly seen malignancy in the eye. Its incidence is second only to retinoblastoma. It has a high degree of malignancy, proliferation, and invasiveness and can metastasize at an early stage [30]. Some studies have pointed out that metastasis, especially the distant metastasis that breaks through the orbit, is an important cause of death [31]. Surgery is still one of the most effective treatments for the disease, but the 5-year survival rate of sufferers remains not optimistic. However, more than 50% of surgical patients have blood metastases, most of which involve the liver, and eventually cause liver failure and death. Surgical resection did not significantly improve the patient's quality of life and did not achieve the effect of radical treatment of the tumor [32, 33]. Therefore, further research of the molecular causal link of the occurrence and metastasis of UM and the search for tumor molecular markers and new therapeutic targets have important significance and clinical application value.

Apoptosis, that is, programmed CD, can happen through the external pathway of the cell death receptor mediator or the internal pathway of the mitochondrial mediator. Many stimuli induce programmed cell death, such as ROS, RNS, hormones, cell-cell interactions, growing factor extraction, antigens, and chemotherapy [34, 35]. The development of cancer is related to decreased apoptosis and cancer cell proliferation [36]. For that reason, apoptotic induction is considered a valid way of tumor treatment. Herein, our team discovered that HAX-1 triggers programmed cell death in a mitochondrial-reliant signal path. In addition, our team also explored the signaling pathways that might exert impacts on the apoptotic events of uveal melanoma cells triggered by HAX-1. As far as we know, the present research is the first to link HAX-1 to uveal melanoma cell lines and shows that HAX-1 mediated mitochondrion-dependent apoptosis is through the AKT/eNOS pathway.

As an antiapoptotic protein, HAX-1 is crucial for cellular protection via suppressing the stimulation of mitochondria and endoplasm reticulum stress-associated apoptosis signal paths [19]. More and more researches have revealed that the expression of HAX-1 is high in a variety of malignancies, affecting tumor cell proliferation, migration, and apoptosis [37]. Deng et al. discovered that the expression of HAX-1 is high in glioma samples and lineage cells and is related to the clinicopathology features and prognoses of glioma; moreover, it promotes the proliferation of glioblastoma cells and inhibits tumor cell apoptosis. [24] Studies have also found that HAX-1 promotes the proliferative, migratory, invasive abilities, and epithelial interstitial transform of liver carcinoma cells. Another research revealed that HAX-1 suppresses the programmed cell death of prostate carcinoma cells via inhibiting the activation of caspase-9 [38]. Nevertheless, the roles and molecular causal link of HAX-l in the occurrence and progression of UM are still unclear. This study was the first to discover that HAX-1 promotes radiation-induced mitochondrion-reliant programmed cell death of UM cells via the AKT/eNOS signal path, inhibits cell proliferation, and has potential clinical application value.

In this study, the TCGA database first analyzed survival differences in patients with uveal melanoma with diverse haX-1 expressing levels. The results showed that the gene expression level of haX-1 was greater in the risk_high_ group in contrast to the risk_low_ group, and sufferers with higher HAX-1 levels displayed an inferior survival time. For that reason, HAX-1 was chosen as an investigation target. There is increasing proofs that the overexpression of HAX-1 occurs in a variety of malignancies, especially affecting proliferation and invasion. We were interested in the roles and causal link of HAX-1 in uveal melanoma, so we used chemically synthesized siRNA to knock out HAX-1 expression in mum-2B and C918 cells. To evaluate the siRNA knockout efficiency, WB was employed to evaluate the siRNA knockout efficiency. We found that HAX-1 siRNA effectively reduced protein expression of HAX-1 in mum-2B and C918 cells. By CCK-8 analysis, our team discovered that cellular proliferative rates of Mum-2B and C918 cells subjected to siRNA treatment were remarkably lower in contrast to those subjected to siRNA treatment. Those outcomes suggest that HAX-1 can facilitate the development of cancer via regulating uveal melanoma cell proliferation. Consistent with this concept, we further found that haX-1 knockdown inhibited cell proliferation in mum-2B and C918 cells. Transwell migration analysis showed that siHAX-1 significantly reduced the cell migration ability in mum-2B and C918 cells in contrast to siRNA control cells. In addition, haX-1 knockout significantly reduced the pellet-forming ability of uveal melanoma cells compared to the control group.

Internal apoptosis induced by mitochondria triggered by death receptors is represented by activation of caspase-9 [39]. In this study, Sihax-1 mediated the activation of Caspase-3/9 and Cytosolic cyt C, and sihax-1 significantly inhibited MMP in the mitochondrial pathway. In addition, Bcl-2 and Bax are tightly associated with programmed cell death as well. Bcl-2 primarily acts as a global mitochondria membrane protein and produces heterosomes with Bax to avoid mitochondria variations during programmed cell death. The outcomes herein revealed that siHAX-1 remarkably decreased the increased expression of Bcl-2 and Bax in mum-2B and C918 cells. Those outcomes suggest that HAX-1 induces uveal melanoma cell apoptosis mainly through mitochondrial dependence.

Finally, this research demonstrates for the first time that HAX-1 triggers uveal melanoma cell apoptosis via mitochondria dependence via the stimulation of PI3K/AKT/eNOS signal path and favorable modulation of Bax, Caspase 3, and Bcl2. The results of this study suggest that haX-1 activates uveal melanoma cells through PI3K/AKT/eNOS by mediating mitochondrial dependent apoptotic pathways that trigger apoptosis, including loss of MMP, transfer of CyT C, and favorable modulation of Bax, Caspase 3, and Bcl2 as key events associated with apoptosis. Those discoveries reveal that PI3K/AKT/eNOS/mitochondrial signal path plays a pivotal role in haX-1 induction of uveal melanoma cell apoptosis.

## Figures and Tables

**Table 1:** Top 20 upregulated genes and top 20 downregulated genes.

Upregulated genes				Downregulated genes			
Gene name	LogFC	*P* value	Adjusted *P* value	Gene name	LogFC	*P* value	Adjust *P* value
HTR2B	3.53	2.55E-07	1.66E-05	SYNPR	-2.70	3.89E-07	2.24E-05
CHAC1	2.74	6.01E-09	1.28E-06	SPP1	-2.50	1.53E-04	1.73E-03
SLC38A5	2.27	1.00E-05	2.22E-04	MSC	-2.28	2.63E-05	4.51E-04
VGF	2.24	5.76E-06	1.47E-04	GSTA3	-2.17	4.39E-05	6.61E-04
TRPV2	2.18	3.95E-07	2.27E-05	PDE3A	-2.00	1.40E-07	1.07E-05
ECM1	2.15	4.35E-07	2.38E-05	HPGD	-1.95	5.84E-06	1.49E-04
AHNAK2	2.13	6.21E-10	3.72E-07	ENPP2	-1.94	2.46E-07	1.61E-05
ISM1	2.11	6.47E-09	1.29E-06	IL12RB2	-1.89	1.79E-07	1.26E-05
IFI27	1.99	4.56E-05	6.83E-04	BEX1	-1.82	4.21E-05	6.39E-04
COL9A3	1.99	1.15E-06	4.79E-05	ROPN1B	-1.80	6.76E-11	1.16E-07
VTN	1.98	2.08E-06	7.09E-05	BCHE	-1.77	9.90E-07	4.25E-05
MYEOV	1.89	1.03E-05	2.27E-04	GPR27	-1.70	6.57E-08	6.48E-06
PSMB9	1.87	3.56E-06	1.04E-04	LNP1	-1.70	5.37E-08	5.63E-06
WARS1	1.83	8.82E-08	7.75E-06	MTUS1	-1.70	2.45E-09	7.87E-07
GRID1	1.83	1.50E-07	1.12E-05	RNF43	-1.69	2.37E-10	1.99E-07
TNFRSF19	1.82	6.11E-06	1.53E-04	CLEC11A	-1.69	5.45E-06	1.42E-04
RARRES2	1.77	1.89E-06	6.60E-05	LIMS2	-1.68	1.19E-06	4.86E-05
PLN	1.76	2.37E-04	2.42E-03	MLIP	-1.67	6.84E-06	1.66E-04
FERMT3	1.76	3.55E-07	2.09E-05	COL11A1	-1.66	1.18E-06	4.86E-05
LAG3	1.76	4.26E-06	1.18E-04	KCNK2	-1.66	8.05E-07	3.65E-05

## Data Availability

The data used to support the findings of this study are available from the corresponding author by request.

## Abbreviations

HAX-1: Hematopoietic matrix-1-related protein X-1
UM: Uveal melanoma
BP: Biological pathways
OS: Overall survival
GO: Gene Ontology
KEGG: Kyoto Encyclopedia of Genes and Genomes
ROC: Receiver operating characteristic
PBS: Phosphate buffer solution
IFN: Interferon
MF: Molecular functions
CC: Cellular components
RNS: Reactive nitrogen species
OM: Outer membrane
CD: Cell death
SICB: Shanghai Institute of Cell Biology
PNC: Penicillin
RT: Room temperature
PFA: Paraformaldehyde
WB: Western blot
DEGs: Differentially expressed genes
K-M: Kaplan-Meier
Cyt c: Cytochrome c
CAS: Chinese Academy of Sciences
SA: Staphylococcus aureus
HCMV: Human cytomegalovirus
FBS: Fetal bovine serum.
